# Navigating Biopsy Safety: Complication Rates Under Ultrasound and CT Guidance

**DOI:** 10.3390/diagnostics15202641

**Published:** 2025-10-20

**Authors:** Theresa Sophie Patzer, Franziska Müller, Michael Meir, Henner Huflage, Lukas Müller, Thorsten Alexander Bley, Jan-Peter Grunz, Andreas Steven Kunz

**Affiliations:** 1 Department of Diagnostic and Interventional Radiology, University Hospital Würzburg, Oberdürrbacher Straße 6, 97080 Würzburg, Germany; 2 Department of Radiology, Massachusetts General Hospital, Harvard Medical School, 32 Fruit Street, YAW 6044, Boston, MA 02114, USA; 3 Department of General, Visceral, Vascular and Pediatric Surgery, University Hospital Würzburg, Oberdürrbacher Straße 6, 97080 Würzburg, Germany; 4 Department of Diagnostic and Interventional Radiology, University Hospital Mainz, Langenbeckstraße 1, 55131 Mainz, Germany

**Keywords:** tomography, X-ray computed, percutaneous biopsy, complications, pneumothorax, bleeding, lung, liver

## Abstract

**Background/Objectives:** The frequency of image-guided biopsies has increased substantially in recent decades; however, high technical success rates are offset by potential complications. **Methods:** This retrospective study compared the safety profile of ultrasound- and CT-guided percutaneous biopsies in 250 patients involving the liver, thoracic organs, retroperitoneum, peripheral lymph nodes, and bone. The parameters analyzed included procedure duration, technical success, as well as type, frequency, severity, timing, and treatment of complications. Statistical comparisons comprised Mann–Whitney-U and Chi-square tests. **Results:** The overall technical success rate was 97.6%, with no significant difference between CT and ultrasound (*p* = 0.491). Ultrasound-guided biopsies were performed more often in women; CT-guided procedures were performed more often in men (*p* = 0.031). Ultrasound-guided interventions were significantly faster with a median duration of 19:00 min vs. 25:30 min in CT (*p* < 0.001). Median radiation dose for CT-guided procedures was 445 mGy·cm (interquartile range 307.8–634.0). Including minor events, complications occurred in 19.6% of cases. Complication rates were significantly higher for CT- (30.3%) compared to ultrasound-guided biopsies (7.6%; *p* < 0.001). Bleeding and pneumothorax were significantly more frequent in CT-guided interventions (*p* = 0.004). Most complications were mild (85.7%) with no life-threatening events. The majority of complications occurred within four hours post-biopsy (93.9%). The severity of complications did not differ significantly between modalities (*p* = 0.399). **Conclusions:** CT-guided biopsies were associated with higher complication rates, likely reflecting procedural complexity and better detection of minor complications. Post-interventional complications such as pneumothorax and bleeding were mostly mild, while severe complications occurrence was extremely rare.

## 1. Introduction

Over the past two decades, image-guided minimally invasive percutaneous biopsies have become an integral component of modern diagnostics. Such interventional radiological procedures enable tissue sampling and are associated with high diagnostic accuracy. Complications are comparatively infrequent, mainly of mild severity and only rarely lead to an extension of the inpatient stay [1]. Compared to surgical excisional biopsies, percutaneous biopsies are less invasive, carry fewer risks with lower mortality and morbidity, shorten hospital stays, and reduce healthcare costs [2]. As a result, they have largely replaced diagnostic surgical procedures, which are only required for inaccessible lesions or cases in which image-guided biopsy yields non-diagnostic results [3]. Ultrasound and CT are the most commonly used modalities for image-guiding biopsies as they are widely available, inexpensive, and able to visualize lesions with high precision. Less frequently, CT fluoroscopy, magnetic resonance imaging, stereotaxis or positron emission tomography CT are employed [4].

Technological advancements in imaging resolution and needle design have further improved the safety, precision, and efficiency of these procedures. According to Cerci et al., the introduction of image guidance increased the success rate of lung biopsy from 40% in 1931, when interventions were still performed in blinded fashion, to 90% in 1937. In addition, the number of complications during the procedure also fell significantly [5]. Ultrasound guidance is often preferred due to its real-time capacity, lack of ionizing radiation, and cost-effectiveness. However, ultrasound has limitations in certain anatomical regions, particularly when acoustic windows are restricted due to bone structures or air-filled organs. In such cases, CT guidance is the method of choice for complex biopsies, despite the associated radiation exposure [6]. CT offers excellent visualization of deep-seated lesions and adjacent structures as well as detection of potential complications. The first CT-guided puncture was performed by Alfidi et al. in 1975 on a retroperitoneal tumor [7]. Since fine-needle biopsies are less frequently diagnostic than core needle biopsies due to the smaller amount of material obtained, core needle biopsy has become the method of choice for determining dignity and classifying tumors. The choice of modality and technique depends on the organ system and the lesion characteristics.

Generally, the location of the lesion to be biopsied ultimately determines the extent to which a lesion is accessible for a sonographic biopsy and whether a CT-guided biopsy is preferable. For instance, obesity represents a limitation for ultrasound guidance. Thoracic biopsies are typically performed under CT guidance. As lung parenchyma is poorly visualized with ultrasound, and precise needle placement is essential, especially in the vicinity of structures at risk as mediastinal structures and vasculature, ultrasound-guided puncture represents a suitable alternative in superficial lesions at most [8,9]. Additionally, the rib cage and aerated lung tissue impede ultrasound transmission, making it difficult to obtain a reliable acoustic window [10]. Furthermore, CT facilitates immediate detection of complications such as hemorrhage and pneumothorax, which are common adverse events following lung biopsy [11]. Similarly, retroperitoneal biopsies are primarily performed under CT guidance due to limited sonographic access caused by overlying musculature and bone structures. Furthermore, long needle paths can occasionally favor the use of CT [12]. Contrarily, in hepatic interventions, ultrasound is oftentimes the preferred modality of choice due to the liver’s favorable anatomical position and echogenicity. CT guidance may be favored for lesions located near the pleura, for relatively distant or poorly accessible lesions [13,14,15]. Bone biopsies are generally performed under CT guidance with high-resolution visualization of osseous lesions [16,17]. In contrast, superficial lymph nodes are easily accessible via ultrasound, which enables real-time monitoring and enhances safety and accuracy [18].

The objective of this study was to evaluate the safety profile of ultrasound- and CT-guided percutaneous biopsies across different organ systems. Particularly, the study aimed to assess the type, frequency, severity, and time frame of complications.

## 2. Materials and Methods

### 2.1. Study Design

This retrospective single-center study was conducted at the radiology department of a tertiary care university hospital and received appropriate approval from the local institutional review board (IRB number: 2023080203, 3rd August 2023). The requirement for informed consent was waived. The investigation was executed in accordance with all applicable laws and regulations. All patients who underwent ultrasound- or CT-guided percutaneous core biopsy between January 2021 through December 2022 were enrolled. A total of 250 patients formed the final study sample. CT-guided biopsies were performed using a single-source CT scanner (Somatom Definition AS, Siemens Healthineers, Erlangen, Germany), while ultrasound-guided interventions were carried out employing a high-end ultrasound system (Acuson Sequoia, Siemens Healthineers). In order to minimize the radiation dose applied, the scan volume for biopsy planning was limited to the target region with selection of a reference layer. For needle guidance, the scan volume was minimized to this reference layer with one adjacent layer on each side. For final evaluation of complications, a spiral was acquired that was just large enough to rule out immediate post-interventional complications. For further radiation dose management, lung biopsies were acquired using a low-dose protocol. Inclusion was independent of the target lesion location or the biopsy origin.

### 2.2. Data Collection

Data were collected retrospectively using the institutional clinic information system. This included analysis of procedural documents, electronic medical reports, pathology findings, and radiology reports. Image analysis was performed employing a dedicated picture archiving and communication system software (Merlin 7.4, Phönix-PACS, Freiburg, Germany) in conjunction with diagnostic monitors certified for clinical readings (RadiForce RX660, EIZO, Hakusan, Ishikawa, Japan).

The following parameters were recorded: patient sex, patient age at the time of the intervention, and the imaging modality used. For CT-guided biopsies, the dose-length product (DLP (mGy·cm)) was extracted from the individual scan protocol. DLP was reported as standard. Due to variability of conversion factors, an estimated effective dose was not provided. The duration of each procedure was determined by PACS documentation of start and end of intervention.

In addition to the punctured organ, technical success and complications were recorded. Technical success was defined as the correctly performed procedure yielding tissue samples sufficient for histopathological diagnosis [19]. Complications were documented by type, time of occurrence, severity, and treatment required (Table 1). The technical success and the occurrence of complications were noted as binary variables (present or absent). Timing of complications was categorized into four intervals: complications that occurred within 0–4 h after the procedure, between 4 and 12 h after the biopsy, between 12 and 24 h after the procedure, or more than 24 h after the biopsy. Four different treatment approaches were differentiated regarding complications: no specific therapy, conservative or symptom-oriented treatment, radiological-interventional treatment, or surgical treatment. Only two complication types, bleeding and pneumothorax, were documented within the patient sample. Complication severity was classified using a modified version of the Society of Interventional Radiology adverse event classification system (SIR AE). Due to the retrospective nature of the study and associated limitations in data availability, only part A and sub-item A of part B were applied [20]. In summary, these are defined as follows: Part A (adverse event description) consists of sub-item A, a narrative description of the adverse event, and sub-item B, an assessment of adverse event severity, with severity levels defined as 1 = mild, 2 = moderate, 3 = severe, 4 = life-threatening or disabling, and 5 = patient death. Sub-item A of part B, regarding the causality, is subdivided into three categories; Category 1: AE not caused by the procedure, Category 2: Unknown whether AE was caused by the procedure and Category 3: AE caused by the procedure (Table 2). For further statistical analyses, target organs were grouped according to similar complication profiles during biopsy: The group “thoracic organs” includes both lung biopsies and biopsies of the mediastinal lymph nodes. Retroperitoneal structures, mainly lymph nodes, paraaortic lymph nodes and kidney biopsies were summed up as “retroperitoneum”. The “peripheral lymph nodes” group includes axillary, iliac, inguinal, pelvic and cervical lymph nodes. Additionally, “liver” and “bone” were defined as singular groups.

### 2.3. Biopsy Procedure

Depending on the location of the lesion to be addressed by biopsy, ultrasound and/or CT examinations were performed to plan the biopsy, select a suitable modality and identify the ideal access route. The choice of guidance modality (CT or ultrasound) was determined at the discretion of the interventionalist. All procedures were executed under local anesthesia by board-certified radiologists with at least 5 years of training in the field. The amount of local anesthetic (mepivacaine, Mecain 1%, PUREN Pharma GmbH & Co. KG, Munich, Germany) varied depending on the location, access route and intensity of the pain, with 10 mg usually being administered subcutaneously. In individual cases, additional sedation (typically propofol) was administered by colleagues from anesthesiology in order to perform the biopsy safely and adequately in patients experiencing pain and restlessness. Following high-risk biopsies, such as liver biopsies, hemoglobin levels were monitored, and in the case of lung biopsies, a post-procedural X-ray of the lungs was ordered after four hours. In addition, patients were required to remain in bed for four hours after the procedure and were monitored clinically on the ward.

Patients were either placed in supine, lateral or prone position in order to facilitate an ideal access and good practicability. CT-guided biopsies were performed using 17 G coaxial and 18 G biopsy needles. A dedicated biopsy system was used, combining a disposable biopsy needle with a reusable punch gun (BARD^®^ MAGNUM^®^ Punch Gun, Aurosan, Essen, Germany). Penetration depth was adjusted to either 15 or 22 mm. For ultrasound-guided biopsies, 16 G coaxial and 18 G biopsy needles were used in combination with a disposable system (Histocore HC, BIP Biomedizinische Instrumente und Produkte GmbH, Türkenfeld, Germany), varying between an insertion depth of 18 mm and 25 mm. Punch biopsies were performed freehand, employing a semi-automatic punch biopsy needle (Tru-Cut, Merit Medical, South Jordan, UT, USA). Bone biopsies were executed using an 11 G perforating bone biopsy system (Madison, Merit Medical). In the case of persistent bleeding, the access channel was treated post-interventionally with gel foam particles (Gelita-Spon Standard, Asia Benz GmbH, Herrenberg, Germany). To rule out a post-procedural pneumothorax, a chest X-ray was taken in an upright position four hours after thoracic interventions.

### 2.4. Statistical Analysis

All data were analyzed with dedicated statistical software (SPSS Statistics 29.01, IBM, Armonk, NY, USA). Reporting of data comprised median values and interquartile ranges (IQR). Assessment of normal distribution was performed with Shapiro–Wilk tests. Continuous, non-normally distributed variables were compared between ultrasound and CT-guided interventions using Mann–Whitney U tests. Chi-square tests were performed for comparisons of nominal/dichotomous variables. For DLP comparisons between CT-guided interventions, the Kruskal–Wallis test was performed with pairwise post hoc analyses. Statistical significance was assumed for *p* values ≤ 0.05.

## 3. Results

A total of 250 biopsies were performed, of which 132 (52.8%) were CT-guided and 118 (47.2%) ultrasound-guided. The overall technical success was 97.6%, while 98.3% of CT-guided biopsies and 97.0% of ultrasound-guided biopsies were regarded as technically successful. Merely two CT-guided and four ultrasound-guided biopsies were classified as technically unsuccessful. Three liver biopsies were classified as technically unsuccessful with two CT-guided biopsies being interrupted due to a complicated access route and lack of patient compliance. In another case the liver lesion was not sufficiently detected using ultrasound; thus, the interventionalist decided to change modalities with a technically successful CT-guided biopsy being performed. Furthermore, one bone biopsy of the L2 vertebra was technically insufficient due to the size and nature of the lesion. Even after changing the access route, it was not possible to obtain a sufficient biopsy. In addition, a CT-guided biopsy of inguinal lymph nodes was discontinued due to difficult access and the associated increased risk. All biopsy attempts mentioned above were performed without complications. Only one technically unsuccessful CT-guided lung biopsy was associated with a moderate complication. With peri-interventional progredient pneumothorax no sufficient tissue for histopathology could be obtained. After chest drainage placement the pneumothorax rapidly regressed with minimal residual. No adequacy assessment was carried out on site.

No significant difference in the success rates was determined between the modalities (*p* = 0.491).

### 3.1. Age, Sex and Time

The final cohort consisted of 124 males and 126 females with an average age of 60.9 ± 16.3 years (range 6–94 years). No significant difference in age was found between CT-guided and ultrasound-guided interventions (*p* = 0.933). More women underwent ultrasound-guided biopsy, while more men received a CT-guided biopsy (*p* = 0.031). Ultrasound-guided interventions were faster than CT-guided interventions (*p* < 0.001). While the interventions took an average of 23 min (IQR 17–29:45 min), regardless of the modality, the average time required for a CT-guided intervention was 25:30 min (21–31 min) versus 19 min (13–26 min) for an ultrasound-guided intervention.

### 3.2. Biopsied Organs

Livers were biopsied most frequently, with 114 interventions equaling 45.6% of all recorded procedures. Thoracic (15.2%), retroperitoneal (13.6%), bone (13.2%), and peripheral lymph node biopsies (12.4%) were performed with similar frequencies. Table 3 shows the distribution of the different organ groups with regard to the selected modality. Of 250 biopsies, 47.2% were performed under ultrasound-control and 52.8% using CT guidance. Certain organ groups were preferentially biopsied using one of the two imaging methods, e.g., most liver biopsies (76.3%), as well as peripheral lymph node biopsies (74.2%) were performed under ultrasound guidance. In contrast, retroperitoneal biopsies (88.2%) and bone biopsies (87.9%) were mostly performed under CT guidance, thus accounting for 22.7% and 22.0% of all CT-guided biopsies. Furthermore, all thoracic interventions were executed using CT. The chi-square test showed a significant correlation between the selected imaging procedure and the biopsied organ (*p* < 0.001).

### 3.3. Radiation Dose for CT-Guided Biopsies

**Figure 1:**
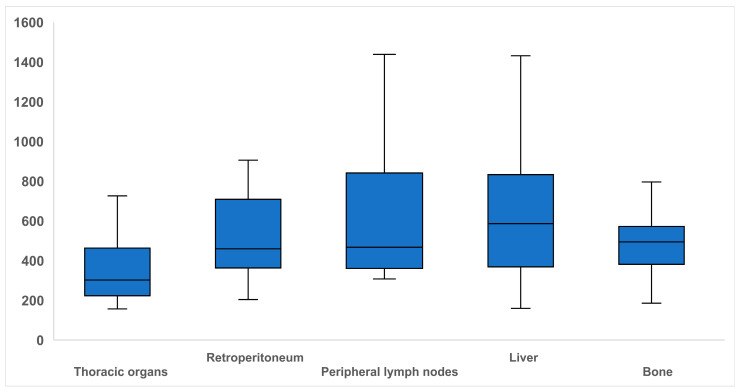
Boxplots illustrating dose-length product in mGy·cm for CT-guided biopsies of different organs. Note—solid line within the box = median; edges of the boxes = upper/lower quartiles; extremes of whiskers = minimum and maximum values within 1.5-fold of interquartile range.

Analyzing the radiation dose for CT-guided biopsies, the median DLP was 445 mGy·cm (307.8–634.0 mGy·cm). The radiation exposure in CT-guided biopsies varied markedly depending on the biopsied organ. The median DLP was highest for liver biopsies at 586 mGy·cm (369.0–833.0) and lowest for thoracic biopsies (303 mGy·cm (223.3–463.0 mGy·cm)). The boxplots in Figure 1 illustrate the DLP distribution according to organs with a significant difference between the groups (*p* < 0.001). Pairwise post hoc comparisons showed significant differences in DLP between thoracic and liver biopsies (*p* = 0.001), as well as between thoracic and retroperitoneal biopsies (*p* = 0.028). No other organ pairs exhibited significant differences (*p* ≥ 0.062).

### 3.4. Complications

Including minor events, complications occurred in 19.6% of all biopsies performed (49/250; 95% CI: 15.2–24.9) with 11.2% representing bleeding and 8.4% pneumothoraces (21/38 = 55.3% of thoracic biopsies). Organ-stratified complication rates were 9.6% for liver (11/114; 5.5–16.5), 65.8% for thoracic (25/38; 49.9–78.8), 20.6% for retroperitoneum (7/34; 10.3–36.8), 9.7% for peripheral lymph nodes (3/31; 3.3–24.9), and 9.1% for bone biopsies (3/33; 3.1–23.6). Within thoracic biopsies, pneumothorax was the most frequent complication, occurring in 52.6% of cases (20/38; 37.3–67.5). Hepatic biopsies were most commonly complicated by bleeding, which occurred in 9.6% (11/114; 5.5–16.5). While complications were documented in 7.6% of ultrasound-guided biopsies, the overall complication rate for CT-guided biopsies was 30.3%, thus CT-guided biopsies were four times more prone to complications than ultrasound-guided biopsies. The Pearson chi-square test indicated a statistically significant association between the chosen imaging modality and the occurrence of complications (*p* < 0.001). Considering the complication rates related to the organ systems, it was found that thoracic biopsies were most prone to complications, 51% of all complications were attributable to thoracic biopsies (n = 25). The lowest complication rates were seen in peripheral lymph node and bone biopsies (n = 3). Organ- and modality-related complication rates are provided in Table 3. Complications that occurred included bleeding of varying severity and pneumothoraces of varying size. Both types of complications were significantly more common in CT-guided interventions (*p* = 0.004). Pneumothoraces occurred exclusively in CT-guided biopsies (n = 21). Evaluating the severity based on a modified version of the new SIR AE classification system, only complications assigned to the first three categories, mild (1), moderate (2), and severe (3), were documented. No life-threatening, disabling (4) or fatal complication (5) were seen. Mild complications were the most common, accounting for 85.7% of the complications. From a total of 49 complications, 81.6% (n = 40) occurred during CT-guided interventions. While moderate (n = 5) and severe (n = 2) complications were documented for CT-guided procedures, only mild events were observed in ultrasound-guided procedures (Table 4).

**Figure 2:**
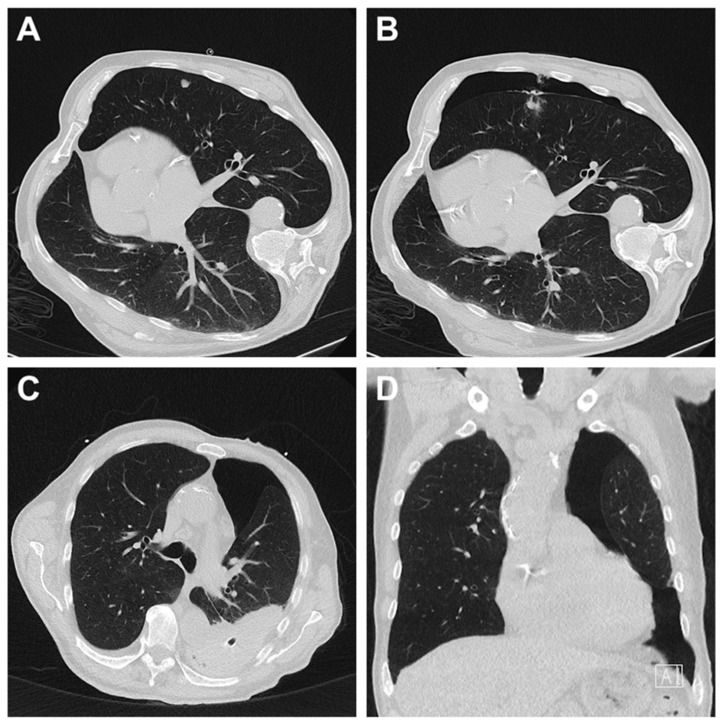
Hemopneumothorax after CT-guided biopsy of a pulmonary nodule in the left upper lobe. In a patient with renal cell carcinoma, CT-guided biopsy of a metastasis-suspected pulmonary nodule in the left upper lobe was performed (**A**). Immediately post-intervention, a pneumothorax measuring up to 4 cm was observed (**B**). 6 days after the intervention, despite the placement of a chest drain, a residual central pneumothorax with a rim width of up to 8 cm remained, accompanied by an organized left-sided hemothorax measuring up to 3 cm, containing multiple air inclusions (**C**,**D**). Due to the persistent hemothorax, video-assisted thoracic surgery for hematoma evacuation was performed.

The chi-square test revealed no significant difference in the severity of complications between the two imaging modalities (*p* = 0.399). If bleeding arose, it was mostly mild (92.9%), there was no case of severe bleeding. In contrast, two severe pneumothoraces occurred (Table 5). Four cases of pneumothorax necessitated chest drainage for treatment, the only case that required surgical intervention was a case of hemopneumothorax where video-assisted thoracic surgery was needed for hematoma evacuation (Figure 2). In most cases (51%), no treatment was necessary.

Overall, 93.9% of complications developed within the first four hours after biopsy. The corresponding chi-square test revealed no significant difference between CT- and ultrasound-guided interventions regarding the timing of complications (*p* = 0.869). Table 6 displays the complications according to time of occurrence and treatment performed.

## 4. Discussion

This study analyzed the safety profile of ultrasound- and CT-guided percutaneous biopsies of the liver, thoracic organs, retroperitoneum, peripheral lymph nodes, and bone. While CT-guided biopsies were four times more likely to result in mainly minor complications than ultrasound-guided procedures (30.3% versus 7.6%), no significant difference was ascertained in the severity of complications. While only minor complications were associated with ultrasound-guided biopsies, moderate (12.5%) and severe (5%) complications occasionally occurred in CT-guided procedures. However, no life-threatening, disabling or fatal complications were observed. No significant difference was seen between modalities in terms of success rate, time of onset of complications and type of treatment of complications. As expected, ultrasound-guided biopsies required significantly less time than CT procedures.

The selection of imaging modality for biopsy guidance is predominantly determined by anatomical and technical factors, including the target organ, the size and location of the lesion, the accessibility, and the modality’s capacity to detect and cope with potential complications. In clinical practice, liver and peripheral lymph node biopsies were preferentially performed under ultrasound guidance, whereas biopsies involving the thorax, retroperitoneum, and the bones were predominantly conducted using CT, consistent with previous reports [21,22,23]. CT is often employed in cases involving difficult access routes and adjacent vulnerable structures, thereby inherently increasing the risk of complications. Moreover, minor complications might be underestimated or missed with ultrasound, as CT allows for better visualization, even of subtle findings. This potential indication bias could be mitigated by matching patient cohorts undergoing biopsies of comparable anatomic complexity and subsequently comparing outcome based on image modality. However, such an approach would only be applicable to lesions that are adequately visualized using both ultrasound and CT. These anatomical and technical challenges are also predictive for longer procedure times, reflecting the complexity of such interventions. The extended duration of CT-guided biopsies can further be explained by the necessity for meticulous planning and precise coaxial needle placement. Unlike ultrasound-guided procedures, which allow real-time visualization, CT guidance requires intermittent imaging to verify needle position after each adjustment, contributing to prolonged intervention duration.

A key consideration in CT-guided interventions is the radiation exposure. In contrast to the radiation-free option of ultrasound, CT-based procedures expose patients to ionizing radiation, an important aspect particularly in individuals undergoing multiple scans, younger patients, and women of childbearing age. In such cases, the potential to reduce or avoid radiation exposure should be critically assessed. Interestingly, gender-specific differences were observed, with women more frequently undergoing ultrasound-guided biopsy and men more often biopsied under CT guidance. This may be attributable to differences in physique or even the interventionist’s subconscious decision against radiation exposure in women of childbearing age. In this study, the median DLP across all CT-guided interventions was 445 mGy·cm. Thoracic biopsies were associated with the lowest radiation exposure (303 mGy·cm), while higher median DLP were observed for liver (586 mGy·cm) and retroperitoneal (459.5 mGy·cm) biopsies. These values were notably lower than those reported by Kloeckner et al., who recorded median DLP of 598 mGy·cm for lung and pleural biopsies, 848 mGy·cm for liver, and 889 mGy·cm for retroperitoneal interventions [24]. Said reduction may be associated with advancements in CT technology and the adoption of optimized low-dose scanning protocols. In particular, ultralow-dose protocols for thoracic imaging are well established in clinical practice and allow for the visualization of pulmonary nodules at substantially reduced radiation dose [25]. Since procedural imaging for needle guidance does not require the spatial or contrast resolution of diagnostic CT, significant dose reductions are feasible. Li et al. demonstrated promising results using a low-dose protocol (100 kVp with tin prefiltration), achieving a mean DPL of just 9.84 mGy·cm for CT-guided percutaneous biopsies [26]. The variation in radiation dose across organ groups can largely be attributed to anatomical and physical factors. Pulmonary lesions typically exhibit high inherent contrast due to surrounding air-filled structures, allowing for adequate visualization at low radiation dose. Conversely, lesions in the liver and retroperitoneum are often located deeper and surrounded by more radiodense tissue, requiring higher radiation exposure for precise localization and safe needle placement [27]. However, it should be noted that reported DLP in this retrospective study design represents a scanner-reported metric derived from the volume CT dose index (CTDIvol) and scan length, referenced to standard phantoms. As such, it does not directly account for individual patient habitus, but only indirectly reflects size-related variations through automatic exposure control settings. Further significant reductions in radiation dose may be achieved through the introduction of modern reconstruction algorithms, spectral filtration, and optimized ultra-low-dose imaging protocols and require additional investigation. Also, future multicenter studies with standardized reporting of dose parameters and patient-specific adjustments are warranted to further evaluate and implement these strategies in clinical practice.

With regard to procedural performance, the technical success rate in this study was 97.6%, exceeding the range of 70 to 96% reported by Gala et al. [17]. No significant difference in technical success was observed between imaging modalities (CT 98.3%, ultrasound 97.0%). Procedural success is influenced by multiple factors, including lesion size and location, the number and quality of samples obtained, and the experience and technical expertise of both the interventionalist and pathologist as well as the technical equipment.

Atwell et al. analyzed the incidence of hemorrhage following 15,181 image-guided percutaneous core biopsies and reported a rate of 0.5% for severe, medically significant or more severe bleeding (grade 3 or higher according to the Common Terminology Criteria for Adverse Events, version 3.0, established by the National Cancer Institute) [28]. In contrast, no severe bleeding events were observed in this cohort. Mild and moderate hemorrhage was seen in 11.2% of cases (n = 28). The results of this study, with only up to moderate bleeding, are thus consistent with the results of previous large studies reporting bleeding rates of 0% to 1.1% after percutaneous biopsy [21,29,30,31,32]. It is noteworthy that many of the aforementioned studies were conducted several years ago, and advances in imaging technology and interventional techniques may have contributed to a further reduction in complication rates in recent years. While Atwell et al. [28] postulated that approximately 20% of major bleeding complications occurred more than 24 after intervention, 93.9% of all complications in our study presented within the first four hours following biopsy. The observation is consistent with the findings of Gala et al. [17], who reported that the majority of complications developed within four to six hours following post-procedure. The high proportion of early complications underlines the need for close monitoring immediately after the biopsy in order to detect and treat possible serious events at an early stage.

Considering the complication rates related to the organ systems, thoracic biopsies were most prone to complications. In concordance with other studies, pneumothorax was the most common complication after thoracic percutaneous biopsy. In the present study, post-procedural pneumothoraces occurred in 8.4% of all biopsies, accounting for 52.6% (n = 20) of thoracic biopsies (n = 38). Most pneumothoraces were classified as mild while moderate pneumothoraces were observed in 14.3% and severe pneumothoraces were seen in 9.5% of cases. Chest tube placement was required in four instances, while one patient required video-assisted thoracic surgery for hematoma evacuation in hemopneumothorax. Despite an overall higher complication rate, the majority of complications were manageable and did not require invasive measures. In comparison, Yoon et al. reported a pneumothorax incidence of 19.4% following biopsy of peripheral lung lesions, with severe adverse events in 3.4% of cases [33]. Other studies have reported pneumothorax rates ranging from 25.9% to 49.3%, with chest tube placement required in 2.1% to 21% of cases [34,35,36,37]. These variations may be attributed to factors such as longer biopsy tract length, smaller lesion sizes, or the biopsy of anatomically challenging lesions [38,39,40]. Furthermore, the question arises as to when a plural dehiscence should be considered a pneumothorax and at what point it qualifies as a complication. To a certain extent, a post-procedural pneumothorax is expected due to the anatomical characteristics of the lung. Weinand et al. reported a pneumothorax rate of 89.4% on immediate post-procedure CT and 100% on chest radiography performed one hour after the intervention [11]. Weinand et al. reported that needle diameter, needle type, access site, and lesion size did not significantly influence the incidence of pneumothorax, whereas a higher number of biopsy samples and longer needle tract distances were identified as relevant risk factors. The evaluation of these parameters was beyond the scope of our study. Discrepancies in pneumothorax rates between studies may therefore reflect differences in procedural settings, patient populations, and the interpretation of pleural dehiscence, as discussed above.

Various techniques aimed at reducing pneumothorax incidence, such as tract sealing with different materials, compression of the puncture site using patient rollover methods, or post-procedural positioning with puncture side down, have demonstrated promising results [41,42,43,44]. Although none of these methods were employed in the present study, severe pneumothoraces were rare, and overall, percutaneous CT-guided thoracic biopsy appears to be a safe diagnostic procedure. Given that transfissural approaches and long access paths are associated with a higher risk of pneumothorax, such routes should be avoided whenever possible. In addition to pneumothorax, rare complications such as air embolism, tumor seeding, infection, hemoptysis, and hemothorax have been reported [45,46]. A meta-analysis by Heerink et al. stated an overall complication rate of 38.8% and a pneumothorax rate of 25.3% [45]. Similarly, Weinand et al. reported an overall complication rate of 31.6% with pneumothorax occurring in 30.9% of the cases and chest tube placement in 12.9% of all patients with pneumothorax [11].

These findings are consistent with data provided by the Society of Interventional Radiology, which reports pneumothorax rates ranging from 25.1% (>18 gauge) to 35.3% (<18 gauge) and chest tube placement rates between 11.7% (>18 gauge) and 16.1% (<18 gauge) [47].

Regarding hepatic biopsies, the overall bleeding rate in this cohort was 9.6%, with only one case classified as moderate bleeding and no instance of severe bleeding. In comparison, the Society of Interventional Radiology reported a bleeding incidence requiring transfusion or intervention in 1.3% of hepatic biopsies [47]. Similarly, McGill et al. documented significant hemorrhage in only 0.24% and fatal bleeding in 0.11% of percutaneous hepatic biopsies [31], while Van der Poorten et al. reported a bleeding rate of 0.83% and a procedure-related mortality of 0.2% [3]. Another study noted an overall complication rate of 5.7% following liver biopsy [14]. In the case of retroperitoneal biopsies, complications occurred in 20.6% of patients in this study, which exceeds the complication rates reported in the literature, ranging from 2.3% to 9.4% [14,48]. This discrepancy may be attributed to differences in study design, sample sizes, or definition of complications. In particular, the inclusion of minor complications in the present analyses may have contributed to the higher observed rate and only mild complications were documented in this study. The vast majority of complications occurred within the first four hours following the intervention, which aligns with existing literature reporting that approximately 91% of pneumothoraces develop during or immediately after intervention [49,50].

Some limitations ought to be mentioned regarding this study. First, this retrospective, monocentric study is limited to a study group of 250 biopsies. Deriving complication rate and estimating the severity of complications from this relatively small sample size introduces uncertainty and limits the generalizability of the findings. A larger, multicenter cohort would be necessary to strengthen the statistical power and improve the robustness of the conclusions. Second, the sample size is insufficient to reliably detect rare severe adverse events or perform statistically viable subgroup analyses. This restricts the ability to explore associations between specific risk factors, e.g., lesion location or access route, and complication rates. Third, although biopsies from different anatomical regions were analyzed separately, the study population remained heterogeneous. This study was executed in a real-word population which reflects clinical practice but introduces variability in baseline characteristics, which may have influenced outcomes. This heterogeneity, combined with the retrospective design, may result in information and documentation bias. Since thoracic, retroperitoneal, and bone biopsies almost exclusively require CT guidance, the apparent higher CT-related risk reflects procedural indication bias. Fourth, complications may have been documented and interpreted differently by various physicians, potentially introducing interobserver variability. Without a standardized classification system or central review, such discrepancies may affect data consistency. In addition, this study recorded and evaluated even minor complications, which may partly explain the comparatively higher complication rates observed, especially in contrast to studies that focused exclusively on clinically significant adverse events. Fifth, although pre-procedural laboratory parameters were reviewed in advance, retrospective data collection does not allow for systematic assessment of all relevant confounders, such as detailed patient comorbidities or individual risk factors, predisposing patients to certain complications. Sixth, this study focused on procedure-related complications and did not assess incidental findings or comorbid conditions identified within the scan volume. Lastly, multicenter studies with large cohorts are mandated to validate the findings, further characterize risk factors, and optimize the safety and diagnostic yield of percutaneous image-guided biopsies.

## 5. Conclusions

With a technical success rate of 97.6%, image-guided percutaneous biopsy appears to be highly effective and safe. CT-guided biopsies were associated with higher complication rates than ultrasound-guided procedures, which was potentially attributable to the greater procedural complexity and improved detection of minor complications on CT. Post-interventional complications such as pneumothorax and bleeding were mostly mild, while the occurrence of severe complications was rare.

## Figures and Tables

**Table 1:** Patient- and intervention-specific parameters.

**Patient and Procedure Data**	**Gender**	male	female			
	**Age (years)**					
	**Imaging Modality**	CT (incl. DLP)	ultrasound			
	**Organ**	liver	thoracic organs (lung, lymph nodes mediastinal)	Retroperitoneum (kidney, retroperitoneal space, paraaortic lymph nodes)	Peripheral lymph nodes (axillary, iliac, inguinal, pelvic, cervical)	bone
	**Duration of the Intervention (min)**					
	**Technical Success**	yes	no			
**Complications**	**Complications**	yes	no			
	**Type**	bleeding	pneumothorax			
	**Time**	0–4 h	4–12 h	12–24 h	>24 h	
	**Severity**	mild	moderate	severe	life-threatening or disabling	patient death or unexpected pregnancy abortion
	**Treatment**	no specific therapy	conservative or symptom-oriented treatment	radiological-interventional treatment	surgical treatment	

Note.—DLP = dose-length product.

**Table 2:** Employed modified version of the Society of Interventional Radiology adverse event classification system (SIR AE).

**Part A (Adverse Event Description)**		
A (description narrative of adverse event)		
B (assessment of adverse event)		
	1. Mild	e.g., minimal venous bleeding from the access channel (treatment with gel foam particles if necessary). Minimal free fluid or hematomas, e.g., adjacent to the needle, subcutaneous, intercostal, retrosternal, perirenal, perihepatic, in the lung parenchyma, extradural. Minimal mediastinal emphysema or discrete pleural effusion/pneumothorax (up to 1.5 cm), adjacent hypoventilation
	2. Moderate	e.g., relevant bleeding or pneumothorax (>1.5 cm) with treatment (e.g., chest drainage) if necessary.
	3. Severe	e.g., cardiac and pulmonary complications, significant drop in oxygen saturation, major bleeding, surgical intervention (e.g., hematoma evacuation).
	4. Life-threatening or disabling event	e.g., cardiopulmonary arrest, shock, organ failure
	5. Patient death or unexpected pregnancy abortion	
**Part B (Adverse Event Analysis)**		
A (Causality of adverse event)		
	Category 1	AE not caused by the procedure
	Category 2	Unknown whether AE was caused by the procedure
	Category 3	AE caused by the procedure

**Table 3:** Biopsied organs, image guidance, and complications.

	Liver	Thoracic Organs	Retroperitoneum	Peripheral Lymph Nodes	Bone	Total
**Ultrasound**	87 (73.7%/76.3%)	0 (0.0%/0.0%)	4 (3.4%/11.8%)	23 (19.5%/74.2%)	4 (3.4%/12.1%)	118 (100%/47.2%)
**CT**	27 (20.5%/23.7%)	38 (28.8%/100.0%)	30 (22.7%/88.2%)	8 (6.1%/25.8%)	29 (22.0%/87.9%)	132 (100%/52.8%)
**Total**	114 (45.6%/100%)	38 (15.2%/100%)	34 (13.6%/100%)	31 (12.4%/100%)	33 (13.2%/100%)	250 (100%)
**No complication**	103 (51.2%/90.4%)	13 (6.5%/34.2%)	27 (13.4%/79.4%)	28 (13.9%/90.3%)	30 (14.9%/90.9%)	201 (100%/80.4%)
**Complication**	11 (22.4%/9.6%)	25 (51.0%/65.8%)	7 (14.3%/20.6%)	3 (6.1%/9.7%)	3 (6.1%/9.1%)	49 (100%/19.6%)
**Total**	114 (45.6%/100%)	38 (15.2%/100%)	34 (13.6%/100%)	31 (12.4%/100%)	33 (13.2%/100%)	250 (100%)

Note—the first percentage refers to the row and the second percentage to the column.

**Table 4:** Type and severity of complications.

	Type of Complications		Severity of Complications			
	Bleeding	Pneumothorax	Mild	Moderate	Severe	Total
**Ultrasound**	9 (100%/32.1%)	0 (0.0%/0.0%)	9 (100%/21.4%)	0 (0.0%/0.0%)	0 (0.0%/0.0%)	9 (100%)
**CT**	19 (47.5%/67.9%)	21 (52.5%/100%)	33 (82.5%/78.6%)	5 (12.5%/100%)	2 (5%/100%)	40 (100%)
**Total**	28 (57.0%/100%)	21 (42.9%/100%)	42 (85.7%/100%)	5 (10.2%/100%)	2 (4.1%/100%)	49 (100%)

Note—the first percentage refers to the row and the second percentage to the column.

**Table 5:** Severity of bleeding and pneumothoraces.

**Bleeding**	Mild	26 (92.9%)
	Moderate	2 (7.1%)
	Severe	0 (0.0%)
**Pneumothorax**	Mild	16 (76.2%)
	Moderate	3 (14.3%)
	Severe	2 (9.5%)

**Table 6:** Timing and treatment of complications.

	Timing of Complications				Treatment of Complications			
	0–4 h	4–12 h	12–24 h	>24 h	No	Conservative or Symptom-Oriented	Radiological-Interventional	Surgical
**Ultrasound**	9 (100%)	0 (0.0%)	0 (0.0%)	0 (0.0%)	5 (55.6%)	4 (44.4%)	0 (0.0%)	0 (0.0%)
**CT**	37 (92.5%)	1 (2.5%)	1 (2.5%)	1 (2.5%)	20 (50.0%)	14 (35.0%)	5 (12.5%)	1 (2.5%)
**total**	46 (93.9%)	1 (2.0%)	1 (2.0%)	1 (2.0%)	25 (51.0%)	18 (36.7%)	5 (10.2%)	1 (2.0%)

## Data Availability

Data is made available upon reasonable request.

## Abbreviations

DLP	dose-length product
IQR	interquartile range
